# FPGA-Based Processor Acceleration for Image Processing Applications

**DOI:** 10.3390/jimaging5010016

**Published:** 2019-01-13

**Authors:** Fahad Siddiqui, Sam Amiri, Umar Ibrahim Minhas, Tiantai Deng, Roger Woods, Karen Rafferty, Daniel Crookes

**Affiliations:** 1 School of Electronics, Electrical Engineering and Computer Science, Queen’s University Belfast, Belfast BT7 1NN, UK; 2 School of Computing, Electronics and Maths, Coventry University, Coventry CV1 5FB, UK

**Keywords:** FPGA, hardware acceleration, processor architectures, image processing, heterogeneous computing

## Abstract

FPGA-based embedded image processing systems offer considerable computing resources but present programming challenges when compared to software systems. The paper describes an approach based on an FPGA-based soft processor called *Image Processing Processor* (IPPro) which can operate up to 337 MHz on a high-end Xilinx FPGA family and gives details of the dataflow-based programming environment. The approach is demonstrated for a *k*-means clustering operation and a traffic sign recognition application, both of which have been prototyped on an Avnet Zedboard that has Xilinx Zynq-7000 system-on-chip (SoC). A number of parallel dataflow mapping options were explored giving a speed-up of 8 times for the *k*-means clustering using 16 IPPro cores, and a speed-up of 9.6 times for the morphology filter operation of the traffic sign recognition using 16 IPPro cores compared to their equivalent ARM-based software implementations. We show that for *k*-means clustering, the 16 IPPro cores implementation is 57, 28 and 1.7 times more power efficient (fps/W) than ARM Cortex-A7 CPU, nVIDIA GeForce GTX980 GPU and ARM Mali-T628 embedded GPU respectively.

## 1. Introduction

With improved sensor technology, there has been a considerable growth in the amount of data being generated by security cameras. In many remote environments with limited communication bandwidth, there is a clear need to overcome this by employing remote functionality in the system such as employing motion estimation in smart cameras  [1]. As security requirements grow, the processing needs will only need to increase.

New forms of computing architectures are needed. In late 70’s, Lamport [2] laid the foundation of parallel architectures exploiting data-level parallelism (DLP) using work load vectorisation and shared memory parallelisation, used extensively in Graphical Processing Units (GPUs). Current energy requirements and limitations of Dennard scaling have acted to limit clock scaling and thus reduce future processing capabilities of GPUs or multi-core architectures [3]. Recent field programmable gate array (FPGA) architectures represent an attractive alternative for acceleration as they comprise ARM processors and programmable logic for accelerating computing intensive operations.

FPGAs are proven computing platforms that offer reconfigurability, concurrency and pipelining, but have not been accepted as a mainstream computing platform. The primary inhibitor is the need to use specialist programming tools, describing algorithms in *hardware description language* (HDL), altough this has been alleviated by the introduction of high-level programming tools such as Xilinx’s Vivado High-level Synthesis (HLS) and Intel’s (Altera’s) compiler for OpenCL. While the level of abstraction has been raised, a gap still exists between adaptability, performance and efficient utilisation of FPGA resources. Nevertheless, the FPGA design flow still requires design *synthesis* and *place-and-route* that can be time-consuming depending on the complexity and size of the design [4,5]. This FPGA design flow is alien to software/algorithm developers and inhibits wider use of the technology.

One way to approach this research problem is to develop adaptable FPGA hardware architecture that enables *edit-compile-run* flow familiar to software and algorithm developers instead of hardware *synthesis* and *place-and-route*. This can be achieved by populating FPGA logic with a number of efficient soft core processors used for programmable hardware acceleration. This underlying architecture will be adaptable and can be programmed using conventional software development approaches. However, the challenge is to build an FPGA solution that is more easily programmed whilst still providing high performance. Whilst FPGA-based processor architectures exist such as Xilinx’s MicroBlaze, Altera’s NIOS and others [6,7,8,9], we propose an *Image Processing Processor* (IPPro) processor [10] tailored to accelerate image processing operations, thereby providing an excellent mapping between FPGA resources, speed and programming efficiency. The main purpose of the paper is to give insights into the multi-core processor architecture built using the IPPro architecture, its programming environment and outline its applications to two image processing applications. Our main contributions are:

- Creation of an efficient, FPGA-based multicore processor which advances previous work [10], [11] and an associated dataflow-based compiler environment for programming a heterogeneous FPGA resource comprising it and ARM processors.
- Exploration of mapping the functionality for a *k*-means clustering function, resulting in a possible speedup of up to 8 times that is 57, 28 and 1.7 times more power efficient (fps/W) than ARM Cortex-A7 CPU, nVIDIA GeForce GTX980 GPU and ARM Mali-T628 embedded GPU.
- Acceleration of colour and morphology operations of traffic sign recognition application, resulting in a speedup of 4.5 and 9.6 times respectively on a Zedboard.

The rest of paper is organized as follows: Section 2 outlines the various image processing requirements and outlines how these can be matched to FPGA; relevant research is also reviewed. System requirements are outlined in Section 3 and the soft core processor architecture is also briefly reviewed in Section 4. The system architecture is outlined in Section 5. Experiments to accelerate a *k*-means clustering algorithm and a traffic sign recognition example, are presented in Section 6 and Section 7 respectively. Conclusions and future work are described in Section 8.

## 2. Background

Traditionally, vision systems have been created in a centralized manner where video from multiple cameras is sent to a central back-end computing unit to extract significant features. However, with increasing number of nodes and wireless communications, this approach becomes increasingly limited, particularly with higher resolution cameras [12]. A distributed processing approach can be employed where data-intensive, front-end preprocessing such as sharpening, object detection etc. can be deployed remotely, thus avoiding the need to transmit high data, video streams back to the server.

### 2.1. Accelerating Image Processing Algorithms

Nugteren et al. has characterized image processing operations based on the computation and communication patterns [13] as highlighted in Table 1. The vision processing architecture can be composed of general and special purpose processors, FPGAs or combinations thereof. FPGAs offer opportunities to exploit the fine/coarse grained parallelism that most of the image processing applications exhibit at front-end processing. Heterogeneous architectures comprising CPUs and FPGA fabrics thus offer a good balance in terms of performance, cost and energy efficiency.

Brodtkorb et al. has compared architectural and programming language properties of heterogeneous architectures comprising CPU, GPU and FPGA [14] showing that FPGAs deliver a better performance/W ratio for fixed-point operations; however, they are difficult to program. Different design approaches have been adopted by the research community to build FPGA-based hardware accelerators. These include:

- **Customised hardware accelerator designs in HDLs** which require long development times but can be optimised in terms of performance and area.
- **Application specific hardware accelerators** which are generally optimized for a single function, non-programmable and created using IP cores.
- Designs created using **high-level synthesis tools** such as Xilinx’s Vivado HLS tool and Altera’s OpenCL compiler which convert a C-based specification into an RTL implementation synthesizable code [15] allowing pipelining and parallelization to be explored.
- **Programmable hardware accelerator** in the form of vendor specific soft processors such as Xilinx’s Microblaze and Altera’s NIOS II processors and customized hard/soft processors.

### 2.2. Soft Processor Architectures

Numerous FPGA multiprocessor architectures have been created to accelerate applications. Strik et al. used a heterogeneous multiprocessor system with a reconfigurable network-on-chip to process multiple video streams concurrently in real-time [16]. VectorBlox MXP [7] is the latest of a series of vector-based soft core processor architectures designed to exploit DLP by processing vectors. Optimizations employed include replacing a vector register file with a scratchpad memory to allow for arbitrary data packing and access, removing vector length limits, enabling sub-word single-instruction, multiple-data (SIMD) within each lane and a DMA-based memory interface.

Zhang et al. has created composable vector units [17] and allows a vector program of a dataflow graph (DFG) to be statically compiled and clusters of operations to be composed together to create a new streaming instruction that uses multiple operators and operands. This is similar to traditional vector chaining but is not easily extended to support wide SIMD-style parallelism. The reported speed-ups were less than a factor of two. Further optimizations have been employed in a custom SVP Bluespec [18] where they compared a custom pipeline to the SVP implementation and found that performance was within a factor of two given similar resource usage. Kapre et al. has proposed a GraphSoC custom soft processor for accelerating graph algorithms [19]. It is a three-stage pipelined processor that supports graph semantics (node, edge operations). The processor was designed with Vivado HLS. Each core uses nine BRAMs and runs at 200 MHz.

Octavo [20] is a multi-threaded, ten-cycle processor that runs at 550 MHz on a Stratix IV, equivalent to the maximum frequency supported by memory blocks. A deep pipeline is necessary to support this high operating frequency, but suffers from the need to pad dependent instructions to overcome data hazards. The authors sidestep this issue by designing Octavo as a multi-processor, thus dependent instructions are always sufficiently far apart and NOP padding is not needed. Andryc et al. presented a GPGPU architecture called FlexGrip [8] which like vector processors, supports wide data parallel, SIMD-style computation using multiple parallel compute lanes, provides support for conditional operations, and requires optimized interfaces to on- and off-chip memory. FlexGrip maps pre-compiled CUDA kernels on soft core processors which are programmable and operate at 100 MHz.

## 3. System Implementation

**Figure 1:**
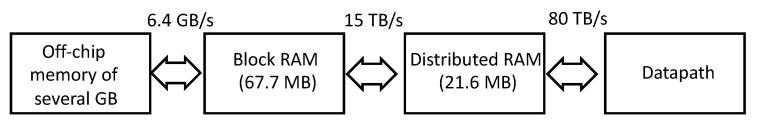
Bandwidth/memory distribution in Xilinx Virtex-7 FPGA which highlight how bandwidth and computation improves as we near the datapath parts of the FPGA.

Whilst earlier versions of FPGAs just comprised multiple Lookup Tables (LUT) connected to registers and accelerated by fast adders, FPGAs now comprise more coarse-grained functions such as dedicated, full-custom, low-power DSP slices. For example, the Xilinx DSP48E1 block comprises a 25-bit pre-adder, a 25 × 18-bit multiplier and a 48-bit adder/subtracter/logic unit, multiple distributed RAM blocks which offer high bandwidth capability (Figure 1), and a plethora of registers which supports high levels of pipelining.

Whilst FPGAs have been successfully applied in embedded systems and communications, they have struggled as a mainstream computational platform. Addressing the following considerations would make FPGAs a major platform rival for “data-intensive” applications:

- *Programmability*: there is a need for a design methodology which includes a flexible data communication interface to exchange data. Intellectual Property (IP) cores and HLS tools [15]/ OpenCL design routes increase programming abstraction but do not provide the flexible system infrastructure for image processing systems.
- *Dataflow support*: the dataflow model of computation is a recognized model for data-intensive applications. Algorithms are represented as a directed graph composed of nodes (actors) as computational units and edges as communication channels [21]. While the actors run explicitly in parallel decided by the user, actor functionality can either be sequential or concurrent. Current FPGA realizations use the concurrency of the whole design at a higher level but eliminate reprogrammability. A better approach is to keep reprogrammability while still maximizing parallelism by running actors on simple “pipelined” processors; the actors still run their code explicitly in parallel (user-specified).
- *Heterogeneity*: the processing features of FPGAs should be integrated with CPUs. Since dataflow supports both sequential and concurrent platforms, the challenge is then to allow effective mapping onto CPUs with parallelizable code onto FPGA.
- *Toolset availability*: design tools created to specifically compile user-defined dataflow programs at higher levels to fully reprogrammable heterogeneous platform should be available.

### High-Level Programming Environment

The proposed methodology employs a reprogrammable model comprising multi-core processors supporting SIMD operation and an associated inter-processor communication methodology. A dataflow design methodology has chosen as the high-level programming approach as it offers concurrency, scalability, modularity and provides data driven properties, all of which match the design requirements associated with image processing systems. A dataflow model allows algorithms to be realized as actors with specific firing rules that are mapped into directed graphs where the nodes represent computations and arcs represent the movement of data. The term data-driven is used to express the execution control of dataflow with the availability of the data itself. In this context, an actor is a standalone entity, which defines an execution procedure and can be implemented in the IPPro processor. Actors communicate with other actors by passing data tokens, and the execution is done through the token passing through *First-In-First-Out* (FIFO) units. The combination of a set of actors with a set of connections between actors constructs a network, which maps well to the system level architecture of the IPPro processors. An earlier version of the programming environment has been is detailed in [11] allowing the user to explore parallel implementation and providing the necessary back-end compilation support.

In our flow, every processor can be thought of as an actor and data is fired through the FIFO structures but the approach needs to be sensitive to FPGA-based limitations such as restricted memory. *Cal Actor Language* (CAL) [22] is a dataflow programming language that has been focussed at image processing and FPGAs and it offers the necessary constructs for expressing parallel or sequential coding, bitwise types, a consistent memory model, and a communication between parallel tasks through queues. RVC-CAL is supported by an open source dataflow development environment and compiler framework, Orcc, that allows the trans-compilation of actors and generates equivalent code depending on the chosen back-ends [23]. An RVC-CAL based design is composed of a dataflow network file (.xdf file) that supports task and data-level parallelism.

**Figure 2:**
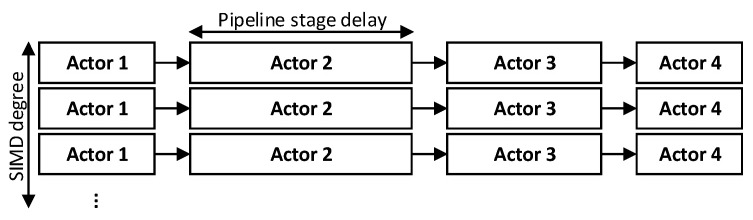
Illustration of possible data and task parallel decomposition of a dataflow algorithm found in image processing designs where the numerous of rows indicate the level of parallelism.

Figure 2 illustrates the possible pipelined decomposition of dataflow actors. These dataflow actors need to be balanced as the worst-case execution time of the actor determines the overall achievable performance. Data-level parallelism is achieved by making multiple instances of an actor and requires SIMD operations that shall be supported by the underlying processor architecture. In addition, it requires software configurable system-level infrastructure that manages control and data distribution/collection tasks. It involves the initialisation of the soft core processors (programming the decomposed dataflow actor description), receiving data from the host processor, distributing them to first-level actors, gathering processed data from the final-level actors and send it back to host processor.

Data-level parallelism directly impacts the system performance; the major limiting factor is the number of resources available on FPGA. An example pipeline structure with an algorithm composed of four actors each having different execution times, and multiple instances of the algorithm realised in SIMD fashion is shown in Figure 2. The performance metric, frames-per-second (fps) can be approximated using $N_{\left(total\_pixels\right)}$ the number of pixels in a frame, $N_{\left(pixel\_consumption\right)}$ the number of pixels consumed by an actor in each iteration and $f_{\left(processor\right)}$ is operating frequency of processor.

(1) $$\mathrm{fps}\approx \frac{f_{\left(processor\right)}*N_{\left(pixel\_consumption\right)}}{N_{\left(total\_pixels\right)}}$$

To improve the fps, the following options are possible:

- *Efficient FPGA-based processor design* that operates at higher operating frequency $f_{\left(processor\right)}$.
- *Reducing the actor’s execution time* by decomposing it into multiple pipelined stages, thus reducing $t_{\left(actor\right)}$ to improve the fps. Shorter actors can be merged sequentially to minimise the data transfer overhead by localising data into FIFOs between processing stages.
- *Vertical scaling to exploit data parallelism* by mapping an actor on multiple processor cores, thus reducing ($n*\frac{N_{\left(total\_pixels\right)}}{N_{\left(pixel\_consumption\right)}}$) at the cost of additional system-level data distribution, control, and collection mechanisms.

**Figure 3:**
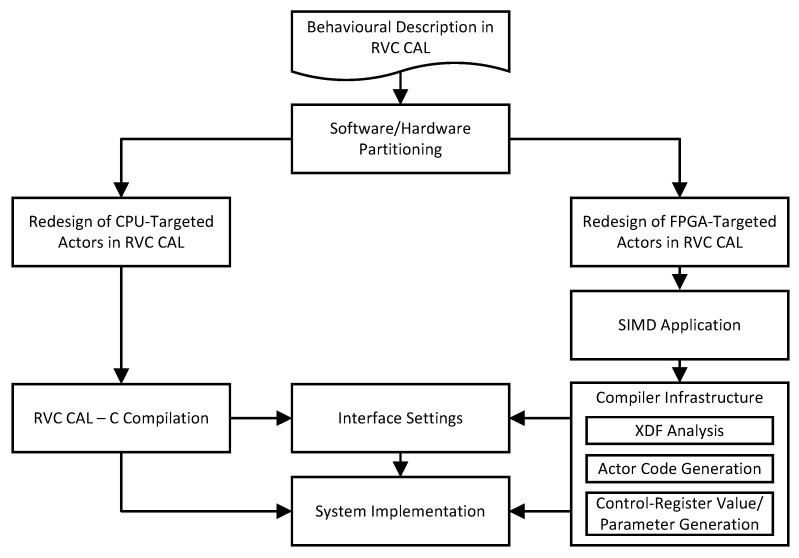
A brief description of the design flow of a hardware and software heterogeneous system highlighting key features. More detail of the flow is contained in reference [11].

The developed tool flow (Figure 3) starts with a user-defined RVC-CAL description composed of actors selected to execute in FPGA-based soft cores with the rest to be run in the host CPUs. By analyzing behaviour, software/hardware partitioning is decided by two main factors, the actors with the worse execution time (determined exactly by number of instructions and the average waiting time to receive the input tokens and send the produced tokens), and the overheads incurred in transferring the image data to/from the accelerator. The behavioural description of an algorithm could be coded in different formats:

- No explicit balanced actors or actions are provided by the user.
- The actors include actions which are balanced without depending on each other, e.g., no global variables in an actor is updated by one action and then used by the other ones; otherwise, these would need to be decomposed into separate actors.
- The actors are explicitly balanced and only require hardware/software partitioning.

There are two types of decomposition, “row-” and “column-wise”. The newly generated data- independent actors can be placed row-wise at the same pipeline stage; otherwise they can be placed column-wise as consecutive pipeline stages. Row-wise is preferred as the overhead incurred in token transmission can be a limiting factor but typically a combination is employed.

If the actors or actions are not balanced, then they need to be decomposed. This is done by detecting a sequence of instructions without branches (unless this occurs at the end) and then breaking the program into basic blocks. The “balance points” whereby the actor needs to be divided into multiple sets of basic blocks such that if each set is placed in a new actor, then need to be found; this will ensure that the overhead of transferring tokens among the sets will not create a bottleneck and infer the selection and use of one with the lowest overhead (See Ref. [11]). Once the graph is partitioned, the original xdf file no longer represents the network topology, so each set of actors must be redesigned separately and their input/output ports fixed and a new set of xdf dataflow network description files, generated. The actors to run on the host CPU are compiled from RVC-CAL to C using the C backend of Orcc development environment, whereas the FPGA-based functionality is then created using the proposed compiler framework.

The degree of SIMD applied will affect the controller interface settings. For a target board, the design will have a fixed number of IPPro cores realized and interconnected with each other and controllers, determined by the FPGA resources and fan-out delay; for the Zedboard considered here, 32 cores are selected. The compilation infrastructure is composed of three distinctive steps:

- Examination of the xdf dataflow network file and assignment and recording of the actor mapping to the processors on the network.
- Compilation of each actor’s RVC-CAL code to IPPro assembly code.
- Generation of control register values, mainly for AXI Lite Registers, and parameters required by the developed C-APIs. running on the host CPU

While FPGA-targeted actor interaction is handled by the compiler, the processes for receiving the image data and storing the output in the edge actors need to be developed. Multiple controllers (programmable by the host CPU) are designed to provide the interface to transfer the data to the accelerators, gather the results and transfer them back to the host. With the host CPU running part of the design and setting control registers, and the IPPro binary codes of the other actors loaded to the proper cores on the accelerator, and the interface between the software/hardware sections set accordingly, the system implementation is in place and ready to run.

## 4. Exploration of Efficient FPGA-Based Processor Design

Image processing applications extensively use multiply and accumulate operations for image segmentation and filtering which can be efficiently mapped to FPGA. On the FPGA, the dedicated memory blocks are located next to the DSP blocks to minimise any timing delays and it is this that determines the maximum operating frequency $\left(f_{max}\right)$ of the processor. It is one of the reasons that many-core and multi-core architectures use simple, light-weight processing datapaths over complex and large out-of-order processors. However, to maintain the balance among soft processor functionality, scalability, performance and efficient utilisation of FPGA resources remain an open challenge.

**Figure 4:**
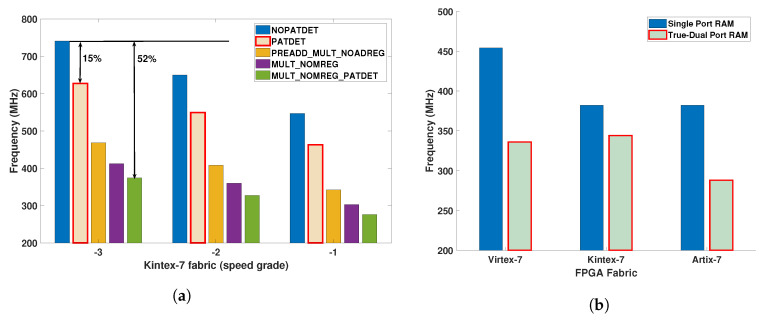
(**a**) Impact of DSP48E1 configurations on maximum achievable clock frequency using different speed grades using Kintex-7 FPGAs for fully pipelined with no (NOPATDET) and with (PATDET) PATtern DETector, then multiply with no MREG (MULT_NOMREG) and pattern detector (MULT_NOMREG_PATDET) and a Multiply, pre-adder, no ADREG (PREADD_MULT_NOADREG) (**b**) Impact of BRAM configurations on the maximum achievable clock frequency of Artix-7, Kintex-7 and Virtex-7 FPGAs for single and true-dual port RAM configurations.

 Figure 4 presents the impact of different configurations of DSP48E1 and BRAM on $f_{max}$ and the parameters required by the developed C-APIs running on the host CPU using different FPGAs. The DSP48E1 has five configurations that offer different functionalities (multiplier, accumulator, pre-adder and pattern detector) based on different internal pipeline configurations that directly impacts $f_{max}$. It varies 15–52% for the same speed grade and reduces by 12–20% when the same design is ported from −3 to −1 speed grade. Configuring the BRAM as a single and true-dual port RAM,  Figure 4b has been created to show that a true-dual port RAM configuration gives a reduction of 25% in $f_{max}$. However an improvement of 16% is possible by migrating the design from Artix-7 to Kintex-7 FPGA technology.

Table 2 shows the distribution of compute (DSP48E1) and memory (BRAM) resources, and highlights the raw performance in GMAC/s (giga multiply-accumulates per second) across the largest FPGA devices covering both standalone and Zynq SoC chips. A BRAM/DSP ratio metric is reported to quantify the balance between compute and memory resources. In Zynq SoC devices, it is higher than standalone devices because more memory is required to implement substantial data buffers to exchange data between FPGA fabric and the host processor, while it is close to unity for standalone devices. This suggests that BRAM/DSP ratio can be used to quantify area efficiency of FPGA designs.

### 4.1. Exploration of FPGA Fabric for Soft Core Processor Architecture

A system composed of light-weight and high-performance soft core processors that supports modular computation with fine and coarse-grained functional granularity is more attractive than fixed dedicated hardware accelerators. A lightweight, soft core processor allows more programmable hardware accelerators to be accommodated onto a single SoC chip which would lead to better acceleration possibilities by exploiting data and task-level parallelism.

**Figure 5:**
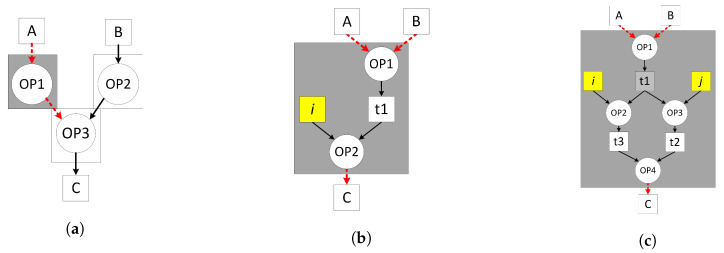
A range of dataflow models taken from [24,25]. (**a**) DFG node without internal storage called configuration ①; (**b**) DFG actor without internal storage t1 and constant i called configuration ②; (**c**) Programmable DFG actor with internal storage t1, t2 and t3 and constants i and j called configuration ③.

**Figure 6:**
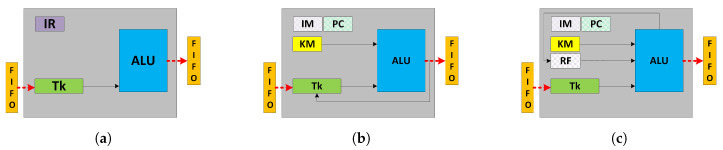
FPGA datapath models resulting from Figure 5. (**a**) Programmable ALU corresponding to configuration ①; (**b**) Fine-grained processor corresponding to configuration ②; (**c**) Coarse-grained processor corresponding to configuration ③.

Gupta et al. [24,25] have reported different dataflow graph models where the functionality corresponds to soft core datapath models ①, ② and ③ as shown in Figure 5. These dataflow models are used to find a trade-off between the functionality of soft core processor and $f_{max}$ and laid the foundation to find the suitable soft core datapath to map and execute the dataflow specification. The input/output interfaces are marked in red while the grey box represents the mapped functionality onto the soft core datapath models as shown in Figure 6.

The first model ① exhibits the datapath of a programmable ALU as shown in Figure 6a. It has an *instruction register* (IR) that defines a DFG node (OP1) programmed at system initialisation. On each clock cycle, the datapath explicitly reads a token from the input FIFO, processes it based on the programmed operation and stores the result into the output FIFO that is then consumed by the following dataflow node (OP3). This model only allows the mapping of data independent, fine-grained dataflow nodes as shown in Figure 5a which limits its applicability due to lack of control and data dependent execution, commonly found in image processing applications where the output pixel depends on the input or neighbouring pixels. This model is only suitable for mapping a single dataflow node.

The second model ② increases the datapath functionality to a fine-grained processor by including BRAM-based *instruction memory* (IM), *program counter* PC and *kernel memory* (KM) to store constants as shown in Figure 6b. Conversely, ② can support mapping of multiple data independent dataflow nodes as shown in Figure 5b. The node (OP2) requires a memory storage to store a variable (t1) to compute the output token (C) which feeds back from the output of the ALU needed for the next instruction in the following clock cycle. This model supports improved dataflow mapping functionality over ① by introducing an IM which comes at the cost of variable execution time and throughput proportional to the number of instructions required to implement the dataflow actor. This model is suitable for accelerating combinational logic computations.

The third model ③ increases the datapath functionality to map and execute a data dependent dataflow actor as shown in Figure 5c. The datapath has memory in the form of a *register file* (RF) which represents a coarse-grained processor shown in Figure 6c. The RF stores intermediate results to execute data dependent operations, implements (feed-forward, split, merge and feedback) dataflow execution patterns and facilitates dataflow transformations (actor fusion/fission, pipelining etc.) constraints by the size of the RF. It can implement modular computations which are not possible in ① and ②. In contrast to ① and ②, the token production/consumption (P/C) rate of ③ can be controlled through program code that allows software-controlled scheduling and load balancing possibilities.

### 4.2. Functionality vs. Performance Trade-Off Analysis

The presented models show that the processor datapath functionality significantly impacts the dataflow decomposition, mapping and optimisation possibilities, but also increases the processor critical path length and affects $f_{max}$ by incorporating more memory elements and control logic.

**Figure 7:**
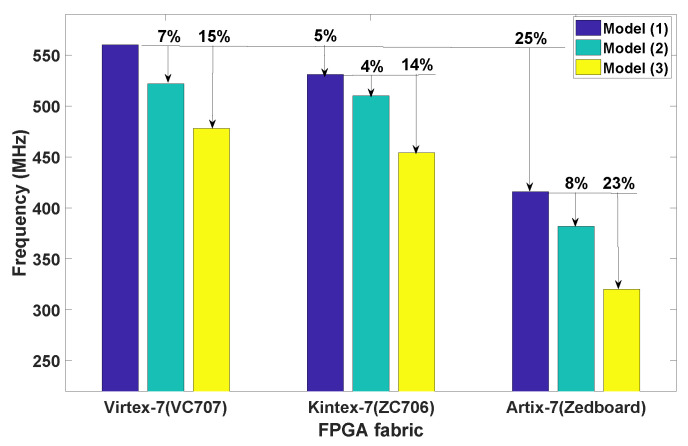
Impact of the various datapath models ①, ②, ③ on $f_{max}$ across Xilinx Artix-7, Kintex-7 and Virtex-7 FPGA families.

Figure 6 shows the datapath models and their memory elements, where the memory resources *(IM, KM, RF)* have been incrementally allocated to each model. Each presented model has been coded in Verilog HDL, synthesised and placed and routed using the Xilinx Vivado Design Suite v2015.2 on Xilinx chips installed on widely available development kits which are Artix-7 (Zedboard), Kintex-7 (ZC706) and Virtex-7 (VC707). The obtained $f_{max}$ results are reported in Figure 7.

In this analysis, $f_{max}$ is considered as the performance metric for each processor datapath model and has a reduction of 8% and 23% for ② and ③ compared to ① using the same FPGA technology. For ②, the addition of memory elements specifically IM realised using dedicated BRAM affects $f_{max}$ by ≈ 8% compared to ①. Nevertheless, the instruction decoder (ID) which is a combinational part of a datapath significantly increases the critical path length of the design. A further 15% $f_{max}$ degradation from ② to ③ has resulted by adding memory elements KM and RF to support control and data dependent execution, which requires additional control logic and data multiplexers. Comparing different FPGA fabrics, a $f_{max}$ reduction of 14% and 23% is observed for Kintex-7 and Artix-7. When ③ is ported from Virtex-7 to Kintex-7 and Artix-7, a maximum $f_{max}$ reduction of 5% and 33% is observed.

This analysis has laid firm foundations by comparing different processor datapath and dataflow models and how they impact the $f_{max}$ of the resultant soft-core processor. The trade-off analysis shows that an area-efficient, high-performance soft core processor architecture can be realised that supports requirements to accelerate image pre-processing applications. Among the presented models, ③ provides the best balance among functionality, flexibility, dataflow mapping and optimisation possibilities, and performance. This model is used to develop a novel FPGA-based soft core IPPro architecture in Section 4.3.

### 4.3. Image Processing Processor (IPPro)

**Figure 8:**
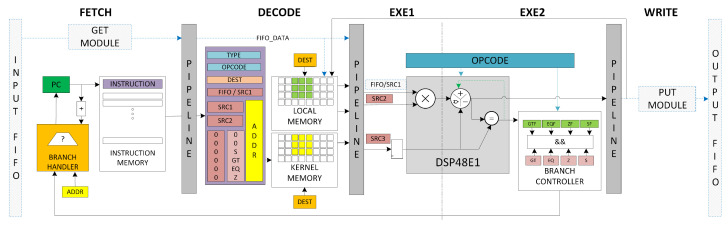
Block diagram of FPGA-based soft core Image Processing Processor (IPPro) datapath highlighting where relevant the fixed Xilinx FPGA resources utilised by the approach.

The IPPro is a 16-bit signed fixed-point, five-stage balanced pipelined RISC architecture that exploits the DSP48E1 features and provides balance among performance, latency and efficient resource utilization [10]. The architecture here is modified to support mapping of dataflow graphs by replacing the previously memory mapped, data memory by stream driven blocking input/output FIFOs as shown in Figure 8. The IPPro is designed as in-order pipeline because: (1) it consumes fewer area resources and can achieve better timing closure leading to the higher processor operating frequency $f_{max}$; (2) the in-order pipeline execution is predictable and simplifies scheduling and compiler development. The datapath supports the identified execution and memory access patterns (Table 1), and can be used as a coarse-grained processing core. IPPro has an IM of size 512 × 32, a RF of size 32 × 16 to store pixels and intermediate results, a KM of size 32 × 16 to store kernel coefficients and constant values, blocking input/output FIFOs to buffer data tokens between a producer, and a consumer to realise pipelined processing stages.

Table 3 outlines the relationship between data abstraction and the addressing modes, along with some supported instructions for the IPPro architecture, facilitating programmable implementation of point and area image processing algorithms. The *stream access* reads a stream of tokens/pixels from the input FIFO using GET instruction and allows processing either with constant values (Kernel Memory-FIFO) or neighbouring pixel values (Register File-FIFO or Register File-Register File). The processed stream is then written to the output FIFO using PUSH instruction. The IPPro supports arithmetic, logical, branch and data handling instructions. The presented instruction set is optimized after profiling use cases presented in [10,26].

The IPPro supports branch instructions to handle control flow graphs to implement commonly known constructs such as if-else and case statements. The DSP48E1 block has a *pattern detector* that compares the input operands or the generated output results depending on the configuration and sets/resets the PATTERNDETECT (PD) bit. The IPPro datapath uses the PD bit along with some additional control logic to generate four flags zero (ZF), equal (EQF), greater than (GTF) and sign (SF) bits. When the IPPro encounters a branch instruction, the branch controller (BC) compares the flag status and branch handler (BH) updates the PC as shown in Figure 8.

The IPPro architecture has been coded in Verilog HDL and synthesized using Xilinx Vivado v2015.4 design suite on Kintex-7 FPGA fabric giving a $f_{max}$ of 337 MHz. Table 4 shows that the IPPro architecture has achieved 1.6–3.3× times higher operating frequency ($f_{max}$) than the relevant processors highlighted in Section 2.2 by adopting the approach presented in Section 4. Comparing the FPGA resource usage of Table 4, the flip-flop utilisation (FF) is relatively similar except for the FlexGrip which uses 30× more flip-flops. Considering LUTs, the IPPro uses 50% less LUT resources compared to MicroBlaze and GraphSoC. To analyse design efficiency, a significant difference (0.76–9.00) in BRAM/DSP ratio can be observed among processors. Analysing design area efficiency, a significant difference 0.76–9.00 in BRAM/DSP ratio is observed which makes IPPro an area-efficient design based on the proposed metric.

### 4.4. Processor Micro-Benchmarks

A commonly used performance metric for a processor is the time required to accomplish a defined task. Therefore, a set of commonly used micro-benchmarks [9,27] has been chosen and implemented on the IPPro and compared against a well-established MicroBlaze soft core processor as shown in Table 5a. Each of the chosen micro-benchmarks are fundamental kernels of larger algorithms and often the core computation of more extensive practical applications. The micro-benchmarks were written in standard C and implemented using Xilinx Vivado SDK v2015.1 Xilinx, San Jose, CA, USA. MicroBlaze has been configured for performance with no debug module, instruction/data cache and single AXI-Stream link enabled to stream data into the MicroBlaze using *getfsl* and *putfsl* instructions in C, equivalent to (GET and PUT) in assembly.

Table 5a reports the performance results of the micro-benchmarks and Table 5b shows the area utilisation comparison of the IPPro and the MicroBlaze both implemented on the same Xilinx Kintex-7 FPGA. It shows that the IPPro consumes 1.7 and 2.3 times fewer FFs and LUTs respectively than the MicroBlaze. It can be observed that for streaming functions (3 × 3 filter, 5-tap FIR and Degree-2 Polynomial), the IPPro achieved 1.80, 4.41 and 8.94 times better performance compared to MicroBlaze due to support of single cycle multiply-accumulate with data forwarding and get/push instructions in the IPPro processor. However, as the IPPro datapath does not support branch prediction that impacts its performance implementing data dependent or conditional functions (Fibonacci and Sum of absolute differences); thus, the SAD implementation using the IPPro resulted in a 5% performance degradation compared to Microblaze. On the other hand, for memory-bounded functions such as Matrix Multiplication, IPPro performed 6.7 times better than MicroBlaze due to higher frequency.

## 5. System Architecture

**Figure 9:**
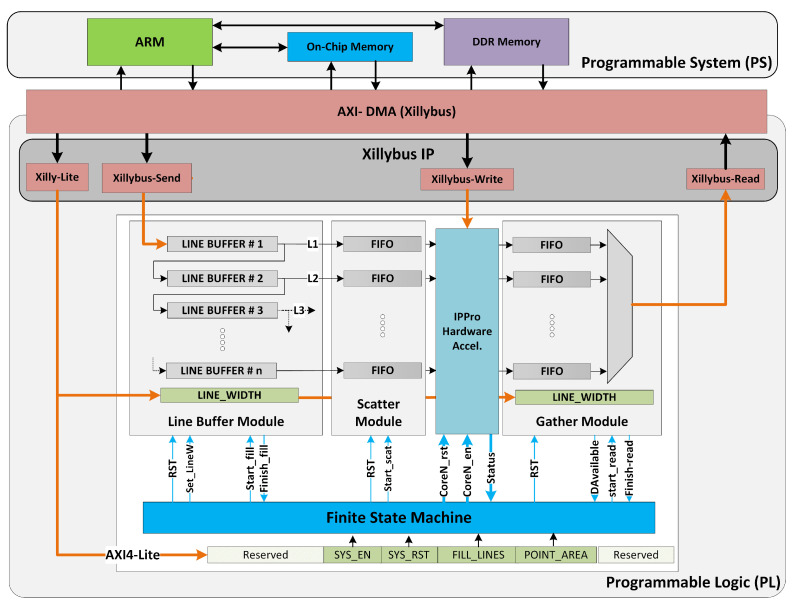
System architecture of IPPro-based hardware acceleration highlighting data distribution and control infrastructure, FIFO configuration and Finite-State-Machine control.

The *k*-means clustering and Traffic Sign Recognition algorithms has been used to explore and analyse the impact of both data and task parallelism using a multi-core IPPro implemented on a ZedBoard. The platform has a Xilinx Zynq XC7Z020 SoC device interfaced to a 256 MB flash memory and 512 MB DDR3 memory. The SoC is composed of a host processor known as programmable system (PS) which configures and controls the system architecture, and the FPGA programmable logic (PL) on which the IPPro hardware accelerator is implemented, as illustrated in Figure 9. The SoC data communication bus (ARM AMBA-AXI) transfers the data between PS and PL using the AXI-DMA protocol and the Xillybus IP core is deployed as a bridge between PS and PL to feed data into the image processing pipeline. The IPPro hardware accelerator is interfaced with the Xillybus IP core via FIFOs. The Linux application running on PS streams data between the FIFO and the file handler opened by the host application. The Xillybus-Lite interface allows control registers from the user space program running on Linux to manage the underlying hardware architecture.

Figure 9 shows the implemented system architecture which consists of the necessary control and data infrastructure. The data interfaces involve stream (Xillybus-Send and Xillybus-Read); uni-directional memory mapped (Xillybus-Write) to program the IPPro cores; and Xillybus-Lite to manage Line buffer, scatter, gather, IPPro cores and the FSM. Xillybus Linux device drivers are used to access each of these data and control interfaces. An additional layer of C functions is developed using Xillybus device drivers to configure and manage the system architecture, program IPPro cores and exchange pixels between PS and PL.

### Control Infrastructure

To exploit parallelism, a configurable control infrastructure has been implemented using the PL resources of the Zynq SoC. It decomposes statically the data into many equal-sized parts, where each part can be processed by a separate processing core. A row-cyclic data distribution [28] has been used because it allows buffering of data/pixels in a pattern suitable for point and area image processing operations after storing them into the line buffers. The system-level architecture (Figure 9) is composed of line buffers, a scatter module to distribute the buffered pixels, a gather module to collect the processed pixels and a finite-state-machine (FSM) to manage and synchronise these modules.

## 6. Case Study 1: *k*-Means Clustering Algorithm

*k*-means clustering classifies a data set into *k* centroids based on the measure e.g., a distance between each data item and the *k* centroid values. It involves: *Distance Calculation* from each data point to the centroids which gives *k* distances and the associated pixels, and a minimum distance is computed from the *k* distance values; *Averaging* where data pixels in the dimension are added up and divided by the number in their dimensions for each cluster, giving an updated centroid value for the following frame. Here we accelerate a functional core of the *k*-means clustering algorithm with 4 centroids to be applied to a 512 × 512 image.

### 6.1. High-Level System Description

The behavioural description is captured in RVC-CAL using Orcc and includes mainly the actor CAL files and the xdf network, derived from .xml format. A dataflow network is constructed with FIFO channels between actors to allow high-throughput passage of tokens from one actor’s output port to another’s input port. The size of FIFO channels can be set. Whilst the length of execution times are the key factor for FPGA acceleration, overheads incurred in transferring the data to/from the PL and accelerators are also important. The SIMD degree was explored by redesigning the FPGA-targeted actors in RVC-CAL and using the compiler to generate the IPPro assembly code. This is done by analysing the xdf file to decide the allocation of actors to the processors and then compiling the function and interconnections.

**Figure 10:**
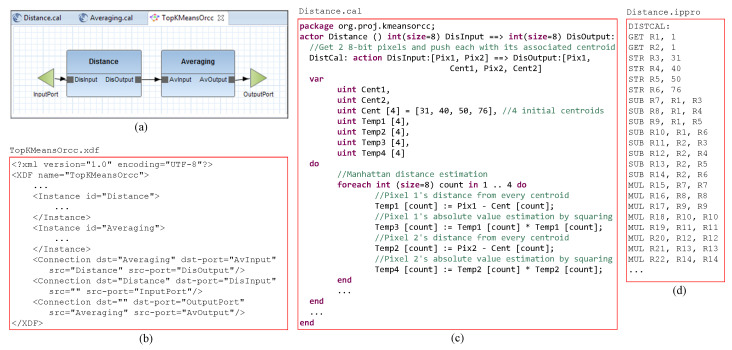
High-level implementation of *k*-means clustering algorithm: (**a**) Graphical view of Orcc dataflow network; (**b**) Part of dataflow network including the connections; (**c**) Part of Distance.cal file showing distance calculation in RVC-CAL where two pixels are received through an input FIFO channel, processed and sent to an output FIFO channel; (**d**) Compiled IPPro assembly code of Distance.cal.

**Figure 11:**
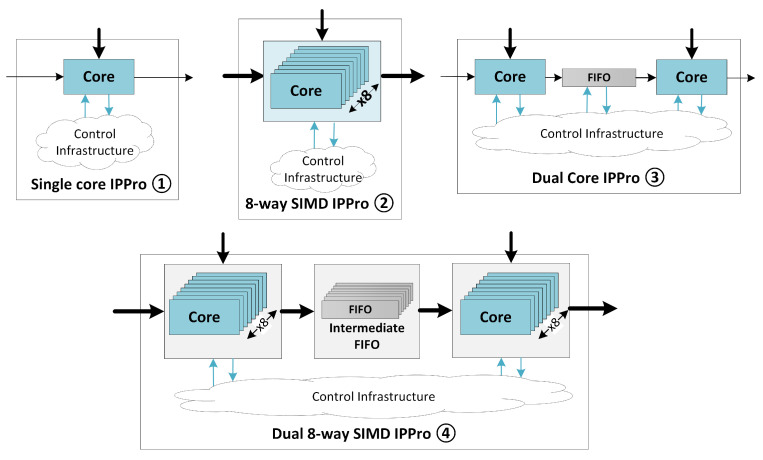
IPPro-based hardware accelerator designs to explore and analyse the impact of parallelism on area and performance based on Single core IPPro ①, eight-way parallel SIMD IPPro ②, parallel Dual core IPPro ③ and combined Dual core 8-way SIMD IPPro called ④.

Every IPPro core sets the hardware units around input/output port connections for the proper flow of tokens, and the compiler is designed to provide the proper signals required by each core. The compiler also generates the setup registers settings and C-APIs parameters, in order to help the controllers distribute the tokens among the cores and gather the produced results. Figure 10 shows the two stages of *k*-means clustering algorithm to be accelerated, and also cores port connections, sample distance calculation code in RVC-CAL and its compiled IPPro assembly code. As Xillybus IP has been used in the system architecture (Section 5), it restricts the clock rate to 100 MHz on Zedboard. To evaluate the IPPro architecture and different dataflow mapping possibilities by exploiting data and task-level parallelism, the *k*-means clustering is accelerated using four acceleration designs listed in Table 6 and illustrated in Figure 11.

### 6.2. IPPro-Based Hardware Acceleration Designs

Table 6 shows the dataflow actor mapping and the exploited parallelism for each design. The block diagram of each IPPro hardware acceleration design is illustrated in Figure 11. Design ① and ② are used to accelerate *Distance Calculation* and *Averaging* stages, where each stage is mapped separately onto individual IPPro cores. To investigate the impact of data and task parallelism, design ③ and ④ are used to accelerate both *Distance Calculation* and *Averaging* stages as shown in Figure 11. The detailed area and performance results are reported in Table 7 and Table 8. The execution time depends on the number of IPPro instructions required to compute the operation and the time require to execute a instruction which corresponds to the operating frequency ($f_{max}$) of IPPro.

Table 7 reports the results obtained by individually accelerating the stages of k-means clustering using ① and ②. In each iteration, the distance calculation takes two pixels and classifies them into one of the four clusters which take an average of 45 cycles/pixel. To classify the whole image, it takes 118.2 ms which corresponds to 8.45 fps. On the other hand, the averaging takes four tokens and produces four new cluster values, which takes an average of 55 clock cycles/pixel results in 145 ms or 6.88 fps. Both the stages involve point-based pixel processing. Therefore design ② was developed and used to exploit data-level parallelism. As a result, the execution time is reduced to 23.32 ms and 27.02 ms for distance calculation and averaging respectively. This is an improvement of 5.1 and 5.4 times over ① (and not the expected 8 times) of the 8-way SIMD implementation (② over ①) as the overhead of data transfer time from/to the accelerator restricts the performance improvement. This came at the cost of 4.1, 2.3 and 8.0 times more BRAMs, LUTs and DSP blocks respectively as reported in Table 8. The major contributor to increased area utilisation is data distribution and control infrastructure.

Table 8 reports the execution time and performance (fps) numbers of both stages together to exploit task and data parallelism using designs ③ and ④. The reported results of ① and ② were obtained by combining the execution time of both stages previously reported in Table 7. Using design ③, the effect of task parallelism implemented via intermediate FIFO results in an average of 63 clock cycles/pixel which is 163 ms (6 fps). By pipelining both actors, ③ has achieved 1.6 times better performance compared to ① at the cost of 1.6 and 2.0 times more BRAM and DSP blocks using the same Xillybus IP infrastructure as ①. The reason for the improvement is the localisation of intermediate data within FPGA fabric using an intermediate FIFO, which hides the data transfer overhead to and from host processor as shown in Figure 11. Investigating the reported area utilisation numbers in Table 8 shows that the area utilisation for design ③ and ④ is not twice as big as ① and ② respectively due to the FPGA resources utilised by the input and output data ports of Xillybus IP. Design ① and ③ requires a single input and output data port, while ② and ④ requires eight input and output data ports. Therefore, a part of FPGA logic used by the Xillybus IP is constant/fixed for ①, ③ and ②, ④.

Analysing the impact of exploiting both task and data-level parallelism using ④ results on average 14 clock cycles/pixel and an execution time of 35.9 ms (2 fps). It is 1.4, 4.5 and 7.3 times better than ②, ③ and ① respectively. For comparison, both stages were coded in C language and executed on an embedded ARM Cortex-A7 processor that achieved execution time of 286 ms (354 fps) which is 8 times slower than the performance achieved by ④.

### 6.3. Power Measurement

This section presents the details of adopted power measurement methods and compares the IPPro-based implementation to the equivalent *k*-means implementation on GPU and CPU. The IPPro power measurements obtained by running post-implementation timing simulation. A *Switch activity interchange format* (SAIF) file is used to record the switching activity of designs data and control signals of each presented IPPro designs. The Xilinx Power Estimator (XPE) takes the SAIF file and reports the power consumption. An equivalent version of *k*-means in CUDA and OpenCL was implemented and profiled on nVIDIA GeForce GTX980 (desktop GPU), ODRIOD-XU3 (Embedded GPU) and ARM Cortex-A7 (CPU) due to in-house availability of both GPU platforms. The nVIDIA desktop GPU card supports 2048 CUDA cores running at a base frequency of 1126 MHz. OpenCL and CUDA were used for programming the GPU, and both stages merged into the single kernel. For performance measurement, OpenCL’s profiling function *clGetEventProfilingInfo* is used which returns the execution time of kernel in nanoseconds. The power consumption during kernel execution was logged using nVIDIA *System Management Interface* (nvidia-smi) which allows to measure the power consumed by the GPU and the host processor separately. It is a command line utility, based on top of the nVIDIA Management Library (NVML), intended to aid the management and monitoring of nVIDIA GPUs.

To set the base line figures and for fair comparison of the FPGA against the GPU technology, an embedded CPU (ARM Cortex-A7) and an embedded GPU (ARM Mali-T628) implementation were carried out on a ODROID-XU3 platform. This is a heterogeneous multi-processing platform that hosts 28 nm Samsung Exynos 5422 application processor which has on-chip ARM Cortex-A7 CPUs and an ARM Mali-T628 embedded GPU. The platform is suitable for power constraint application use cases where the ARM Cortex-A7 CPU and mid-range ARM Mali-T628 GPU runs at 1.2 GHz and 600 MHz respectively. The platform have separate current sensors to measure the power consumption of ARM Cortex-A7 and ARM Mali-T628, thus allowing component-level power measurement capability.

Table 9 shows the results of IPPro-based accelerator designs running on Zedboard where both data and task parallel implementation achieved 4.6 times better performance over task only implementation at the cost of 1.57 times higher power consumption. Table 10 shows the performance results of the *k*-means implementation on Kintex-7 FPGA and compares them against equivalent embedded CPU (ARM Cortex-A7), embedded GPU (ARM Mali-T628) and desktop GPU (nVIDIA GeForce GTX680) implementations in terms of speed (MHz), Power (W) and transistors utilised (TU). The presented embedded CPU results has been considered as a baseline for the comparison.

Both FPGA implementations achieved 6 and 27 times better fps performance than the embedded CPU, whilst the embedded GPU delivered 6.7 times better performance over the FPGA by exploiting parallelism and higher operating frequency. Focusing on the power consumption results, the FPGA consumed 2.1 and 4.9 times less power than both the embedded CPU and embedded GPU respectively. It shows that the FPGA technology delivers a power-optimised solution while the GPU approach provides a performance-optimised solution. Considering the performance and power together, the power efficiency (fps/W) numbers shows that FPGA and embedded GPU implementations are 57 and 33 times more power efficient than embedded CPU and that the FPGA implementation is 24 times more power efficient than embedded GPU. Nevertheless, this power efficiency edge can be further improved by applying dataflow transformations and increasing the number of IPPro cores.

Table 10 compares the FPGA results against desktop GPU and reports resource efficiency as a metric due to significant difference in the power consumption numbers. The resource efficiency has been presented in terms of frames-per-second-per-Transistor-Utilisation (fps/TU) which is 6 and 63 for the 28 nm FPGA and GPU technologies. For embedded CPU and GPU, these results are not reported due to unavailability of transistor count numbers for the ARM. The reported resource efficiency results shows that GPU utilises area resources more efficiently than the FPGA when power is kept out of the equation. Combining all three metrics (fps/W/TU) shows that the advantage gained from FPGA designs is significant i.e., 22 times more efficient than GPU. This advantage becomes more valuable when it is acknowledged that the FPGA-based SoC design is adaptable and allows exploration, profiling and implementation of different dataflow transformation possibilities over dedicated FPGA approaches to accelerate image processing applications for low energy applications.

## 7. Case Study 2: Traffic Sign Recognition

**Figure 12:**
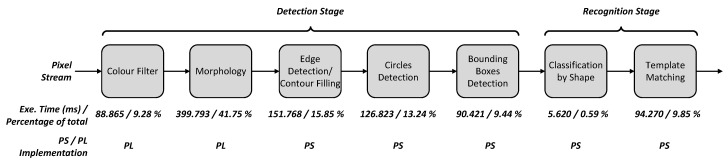
Section execution times and ratios for each stage of the traffic sign recognition algorithm.

Traffic sign recognition is applied in driver assistance systems [29]. In the detection stage, sign candidate areas are extracted from the original image and matched against a list of known templates in the recognition stage. The processing stages along with their execution time and percentage contribution to the overall execution time for 600 × 400 image sign recognition implemented on ARM Cortex-A9 are shown in Figure 12. It involves a colour filter to convert RGB to HSV, morphology filters (erosion and dilation) using 3 × 3 and 5 × 5 circular kernels, edge detection, circles detection to guide the matching process and reduce the number of shapes, bounding box detection to transform the remaining objects into their convex hulls, classification by shape and then template matching. The colour and morphology filters have been chosen for hardware acceleration as they are dominant processing components as shown in Figure 12.

### Acceleration of Colour and Morphology Filter

**Figure 13:**
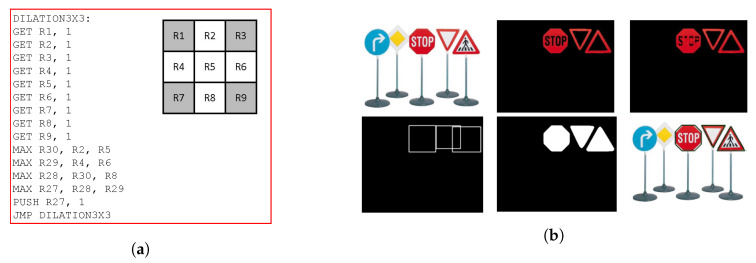
(**a**) The simplified IPPro assembly code of 3 × 3 dilation operation. (**b**) The output result of implemented design.

The IPPro-based hardware accelerators for colour and morphology filter were implemented on Zedboard using the system architecture presented in Section 5 that allows to distribute pixels for *point* and *window* image processing operations. The high-level system description of colour filter actor from RVC-CAL produced program code consists of 160 IPPro assembly instructions. A 3 × 3 circular mask has been used for morphology filter as shown in Figure 13a, to find the maximum (dilation) or minimum (erosion) value in a set of pixels contained within a masked region around the input pixel.

The simplified generated code of RVC CAL-IPPro compilation is shown in Figure 13a. GET and PUSH instructions set the input or output port numbers through which the tokens are received or sent. GET instructions read 9 pixels values and stores them into the register file from R1 to R9. Then, the corner pixels are ignored to impose 3 × 3 circular mask, a maximum value among the remaining pixels max(R1, R4, R5, R6, R8) is computed and stored in R7 to apply dilation operation. This value is then pushed to the output using PUSH instruction. The output result of the implemented design are shown in Figure 13b.

Table 11 presents the results from the Zedboard implementation that has been tested with a set of real images. The hardware accelerated implementation of colour filter stage using 32 IPPro cores reduces the execution time from 88.87 ms down to 19.71 ms compared to software implementation on-chip ARM Cortex-A9. Similarly, the morphology filter stage using 16 IPPro cores has reduced the execution time from 399 ms down to 41.3 ms. The presented IPPro-based hardware acceleration design has achieved a speed-up of 4.5 and 9.6 times over ARM for colour and morphology filters respectively. The achieved speed up for colour filter stage using 32 cores is lower than that of morphology stage using 16 cores, because of the higher number of clock cycles spent on every pixel for colour filter stage; this is due to larger execution time of division coprocessor used for colour filtering.

**Figure 14:**
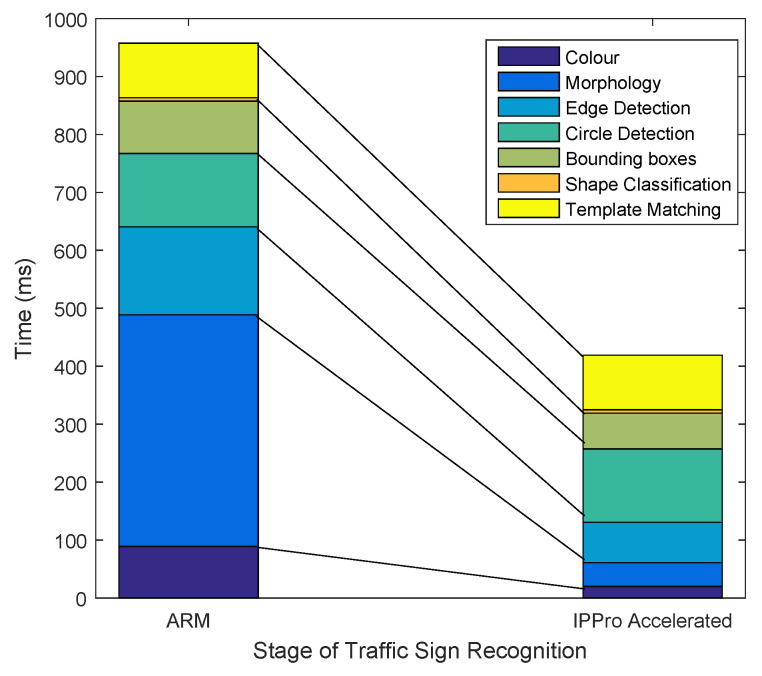
Stage-wise comparison of traffic sign recognition acceleration using ARM and IPPro based approach.

Figure 14 shows the stage-wise acceleration of traffic sign recognition by accelerating colour and morphology filters. Edge/contours detection and bounding boxes stages were improved partially by accelerating the morphology operations. The edge detection is based on the morphology operations by taking the difference between erosion and dilation. Therefore the morphology results obtained by acceleration are further exploited in the host to factor out some operations when doing edge detection.

## 8. Conclusions and Future Work

The paper has presented an FPGA-based hardware acceleration approach for image processing applications using soft core processors which maps efficiently to FPGA resources thereby maintaining performance. By using the DFG model of computations, a design flow has been created which allows the user to partition the design based on processing needs and allows programming of each function. The work has been demonstrated for a *k*-means clustering function and a traffic sign recognition example where maximum speed up of 8 and 9.6 times, respectively, were achieved when compared to software implementation on ARM CPU. For *k*-means clustering, the 16 IPPro cores implementation is 57, 28 and 1.7 times more power efficient (fps/W) than ARM Cortex-A7 CPU, nVIDIA GeForce GTX980 GPU and ARM Mali-T628 embedded GPU. The future work to improve this work is to investigate further dataflow decomposition/mapping optimisations and software-controlled power optimisation techniques such as on-demand enable/disable the IPPro cores.

References

## Figures and Tables

**Table 1:** Categorisation of image processing operations based on their memory and execution patterns [13] allow features of compute and memory patterns to be highlighted and therefore identifying what can be mapped into FPGA.

Operation Type	Domain	Output Depends on	Memory Pattern	Execution Pattern	Examples
Point and Line	Spatial	Single input pixel	Pipelined	One-to-one	Intensity change by factor, Negative image-inversion.
Area/Local	Spatial	Neighbouring pixels	Coalesced	Tree	Convolution functions: Sobel, Sharpen, Emboss.
Geometric	Spatial	Whole frame	Recursive non-coalesced	Large reduction tree	Rotate, Scale, Translate, Reflect, Perspective and Affine.

**Table 2:** Computing resources (DSP48E1) and BRAM memory resources for a range of Xilinx Artix-7, Kintex-7, Virtex-7 FPGA families implemented using 28nm CMOS technology.

Product	Family	Part Number	BRAM (18 Kb Each)	DSP48E1	GMAC/s	BRAM/DSP
Standalone	Artix-7	XC7A200T	730	740	929	0.99
Standalone	Kintex-7	XC7K480T	1910	1920	2845	0.99
Standalone	Virtex-7	XC7VX980T	3000	3600	5335	0.83
Zynq SoC	Artix-7	XC7Z020	280	220	276	1.27
Zynq SoC	Kintex-7	XC7Z045	1090	900	1334	1.21

**Table 3:** IPPro supported addressing modes highlighting the relation to the data processing requirements and the instruction set.

Addressing Mode	Data Abstraction	Supported Instructions
FIFO handling	Stream access	get, push
Register File–FIFO	Stream and randomly accessed data	addrf, subrf, mulrf, orrf, minrf, maxrf etc
Register File–Register File	Randomly accessed data	str, add, mul, mulacc, and, min, max etc.
Kernel Memory–FIFO	Stream and fixed values	addkm, mulkm, minkm, maxkm etc.

**Table 4:** Comparison of IPPro against other FPGA-based processor architectures in terms of FPGA resources used and timing results achieved.

Resource	IPPro	Graph-SoC [19]	FlexGrip 8 SP * [8]		MicroBlaze
FFs	422	551	(103,776/8 =)	12,972	518
LUTs	478	974	(71,323/8 =)	8916	897
BRAMs	1	9	(120/8 =)	15	4
DSP48E1	1	1	(156/8 =)	19.5	3
Stages	5	3	5		5
Freq. (MHz)	337	200	100		211

* Scaled to a single streaming processor.

**Table 5:** Performance comparison of IPPro and MicroBlaze implementations (**a**) Comparison of micro-benchmarks. (**b**) Area comparison.

**a**			
**Processor**	**MicroBlaze**	**IPPro**	
**FPGA Fabric**	Kintex-7		
**Freq (MHz)**	287	337	
**Micro-benchmarks**	**Exec. Time (us)**		**Speed-up**
*Convolution*	0.60	0.14	4.41
*Degree-2 Polynomial*	5.92	3.29	1.80
*5-tap FIR*	47.73	5.34	8.94
*Matrix Multiplication*	0.67	0.10	6.7
*Sum of Abs. Diff.*	0.73	0.77	0.95
*Fibonacci*	4.70	3.56	1.32
**b**			
**Processor**	**MicroBlaze**	**IPPro**	**Ratio**
**FFs**	746	422	1.77
**LUTs**	1114	478	2.33
**BRAMs**	4	2	2.67
**DSP48E1**	0	1	0.00

**Table 6:** Dataflow actor mapping and supported parallelism of IPPro hardware accelerator design presented in Figure 11.

Design	Acceleration Paradigm	Mapping	Parallelism	
			Data	Task
①	Single core IPPro	Single actor	No	No
②	8-way SIMD IPPro	Single actor	Yes	No
③	Dual core IPPro	Dual actor	No	Yes
④	Dual core 8-way SIMD IPPro	Dual actor	Yes	Yes

**Table 7:** Performance measurements for designs ① and ② highlighted in Figure 11.

Single Actor	① Single Core IPPro		② 8-Way SIMD IPPro	
	Exec. (ms)	fps	Exec. (ms)	fps
Distance Calculation	118.21	8.45	23.37	42.78
Averaging	145.17	6.88	27.02	37.00

**Table 8:** Area utilisation and performance results of IPPro-based hardware accelerator designs in Figure 11 exploiting data and task parallelism namely ①, ②, ③ and ④.

*k*-Means Acceleration	Area				Performance	
	LUT	FF	BRAM	DSP	Exec. (ms)	fps
① Combined stages using Single-core IPPro	4736	5197	4.5	1	263.38	3.8
② Combined stages using 8-way SIMD IPPro	10,941	12,279	18.5	8	50.39	19.8
③ Dual-core IPPro	4987	5519	4.5	2	163.2	6
④ Dual 8-way SIMD IPPro	13,864	16,106	18.5	16	35.9	28
Software implementation on ARM Cortex-A7	-	-	-	-	286	3.5

**Table 9:** Power, resource and combined efficiency comparisons of IPPro-based *k*-means clustering implementations on Zedboard (Xilinx Zynq XC7Z020 Artix-7).

	Power (mW)			Freq.	Exec.		Power	TU	Efficiency	
Impl.	Static	Dyn.	Tot.	(MHz)	(ms)	fps	Efficiency	(×$10^{6}$)	(fps/TU)	(fps/W/TU)
							(fps/W)		(×$10^{-8}$)	(×$10^{-9}$)
**③**	118	18	136	100	163.2	6	44.1	591 (9%)	1.0	74.6
**④**	122	92	214	100	35.9	28	130.8	1564 (23%)	1.8	83.6

**Table 10:** Power, resource and combined efficiency comparisons for *k*-means clustering for Xilinx Zynq XC7Z045 Kintex-7 FPGA, nVIDIA GPU GTX980, embedded ARM Mali-T628 GPU and embedded ARM Cortex-A7 CPU.

		Power (W)			Freq.	Exec.		Power	TU	Efficiency	
Plat.	Impl.	Static	Dyn.	Tot.	(MHz)	(ms)	fps	Effic.	(×$10^{9}$)	(fps/TU)	(fps/W/TU)
								(fps/W)		(×$10^{-8}$)	(×$10^{-9}$)
**FPGA**	**③**	0.15	0.03	0.19	337	48.43	21	114.1	0.6 (9%)	3.6	193.1
	**④**	0.16	0.15	0.31	337	10.65	94	300.3	1.0 (6%)	6.0	192.0
**GPU**	**OpenCL**	37	27	64	1127	1.19	840	13.1	1.3 (26%)	63.1	9.8
	**CUDA**	37	22	59	1127	1.58	632	10.7	1.2 (24%)	51.5	8.7
**eGPU**	**Mali**	0.12	-	1.56	600	3.69	271	173	-	-	-
**eCPU**	**Cortex**	0.25	-	0.67	1200	286	3.49	5.2	-	-	-

**Table 11:** IPPro-based acceleration of colour and morphology operations implemented on Zedboard.

Description	Colour	Morphology
No. of cores	32	16
FF	41,624 (39%)	43,588 (41%)
LUT	29,945 (56%)	33,545 (63%)
DSP48E1	32 (15%)	48 (22%)
BRAM	60 (42%)	112 (80%)
Cycles/Pixel	160	26
Exec. (ms)	19.7 (8.7 *)	41.3 (18.3 *)
Speed-up	4.5× (10.3× *)	9.6× (21.75× *)

* The achievable performance using Zynq XC7Z045 Kintex-7.
